# Error bounds for linear complementarity problems of weakly chained diagonally dominant *B*-matrices

**DOI:** 10.1186/s13660-017-1303-5

**Published:** 2017-02-02

**Authors:** Feng Wang

**Affiliations:** College of Science, Guizhou Minzu University, Guiyang, Guizhou 550025 P.R. China

**Keywords:** 90C33, 60G50, 65F35, error bound, linear complementarity problem, weakly chained diagonally dominant matrix, *B*-matrix

## Abstract

In this paper, new error bounds for the linear complementarity problem are obtained when the involved matrix is a weakly chained diagonally dominant *B*-matrix. The proposed error bounds are better than some existing results. The advantages of the results obtained are illustrated by numerical examples.

## Introduction

A linear complementarity problem (*LCP*) is to find a vector $x\in \mathbb{R}^{n\times1}$ such that

$$(Mx+q)^{T}x= 0, \qquad Mx+q\geq0, \quad x\geq0, $$

where $M=[m_{ij}]\in\mathbb{R}^{n\times n}$ and $q\in \mathbb{R}^{n\times1}$. The *LCP* has various applications in the free boundary problems for journal bearing, the contact problem, and the Nash equilibrium point of a bimatrix game [1–3].

The *LCP* has a unique solution for any $q\in \mathbb{R}^{n\times1}$ if and only if *M* is a *P*-matrix [4]. In [5], Chen *et al.* gave the following error bound for the *LCP* when *M* is a *P*-matrix:

$$\bigl\Vert x-x^{*}\bigr\Vert _{\infty}\leq\max _{d\in[0,1]^{n}} \bigl\Vert (I-D+DM)^{-1}\bigr\Vert _{\infty} \bigl\Vert r(x)\bigr\Vert _{\infty}, $$

where $x^{*}$ is the solution of the *LCP*, $r(x)=\min\{x, Mx+q\}$, $D=\operatorname{diag}(d_{i})$ with $0\leq d_{i}\leq1$, and the min operator $r(x)$ denotes the componentwise minimum of two vectors. If *M* satisfies special structures, then some bounds of $\max_{d\in [0,1]^{n}} \Vert (I-D+DM)^{-1}\Vert _{\infty}$ can be derived [6–11].

### Definition 1

[4]

A matrix $M=[m_{ij}]\in\mathbb{R}^{n\times n}$ is called a *B*-matrix if for any $i, j\in\mathbb{N}=\{1,2,\ldots,n\}$,

$$\sum_{k\in N} m_{ik}>0, \qquad \frac{1}{n} \biggl(\sum_{k\in N} m_{ik} \biggr)>m_{ij}, \quad j\neq i. $$

### Definition 2

[12]

A matrix $A=[a_{ij}]\in\mathbb{R}^{n\times n}$ is called a weakly chained diagonally dominant (*wcdd*) matrix if *A* is diagonally dominant, *i.e.*,

$$\vert a_{ii}\vert \geq r_{i}(A)= \sum _{j=1, \neq i}^{n} \vert a_{ij}\vert , \quad \forall i\in\mathbb{N}, $$

and for each $i\notin J(A)=\{ i\in\mathbb{N}: \vert a_{ii}\vert >r_{i}(A)\}\neq\emptyset$, there is a sequence of nonzero elements of *A* of the form $a_{ii_{1}}, a_{i_{1}i_{2}}, \ldots, a_{i_{r} j}$ with $j\in J(A)$.

### Definition 3

[13]

A matrix $M=[m_{ij}]\in\mathbb{R}^{n\times n}$ is called a weakly chained diagonally dominant (*wcdd*) *B*-matrix if it can be written in the form $M=B^{+}+C$ with $B^{+}$ a *wcdd* matrix whose diagonal entries are all positive.

García-Esnaola *et al.* [8] gave the upper bound for $\max_{d\in [0,1]^{n}} \Vert (I-D+DM)^{-1}\Vert _{\infty}$ when *M* is a *B*-matrix: Let $M=[m_{ij}]\in\mathbb{R}^{n\times n}$ be a *B*-matrix with the form

$$M=B^{+}+C, $$

where

1 $$ B^{+}=[b_{ij}]=\left [ \begin{matrix} m_{11}-r_{1}^{+} &\cdots &m_{1n}-r_{1}^{+} \\ \vdots & &\vdots \\ m_{n1}-r_{n}^{+} &\cdots &m_{nn}-r_{n}^{+} \end{matrix} \right ], $$

and $r_{i}^{+}=\max\{0, m_{ij}\vert j\neq i\}$. Then

2 $$ \max_{d\in[0,1]^{n}} \bigl\Vert (I-D+DM)^{-1}\bigr\Vert _{\infty }\leq \frac{n-1}{\min\{\beta,1\}}, $$

where $\beta= \min_{i\in\mathbb{N}}\{\beta_{i}\}$ and $\beta_{i}=b_{ii}-\sum_{j\neq i} \vert b_{ij}\vert $.

To improve the bound in (2), Li *et al.* [14] presented the following result: Let $M=[m_{ij}]\in \mathbb{R}^{n\times n}$ be a *B*-matrix with the form $M=B^{+}+C$, where $B^{+}=[b_{ij}]$ is defined as (1). Then

3 $$ \max_{d\in[0,1]^{n}} \bigl\Vert (I-D+DM)^{-1}\bigr\Vert _{\infty} \leq \sum_{i=1}^{n} \frac{n-1}{\min\{\bar{\beta}_{i},1\}} \prod_{j=1}^{i-1} \Biggl(1+ \frac{1}{\bar{\beta}_{j}} \sum_{k=j+1}^{n} \vert b_{jk}\vert \Biggr), $$

where $\bar{\beta}_{i}=b_{ii}-\sum_{j= i+1}^{n} \vert b_{ij}\vert l_{i}(B^{+})$, $l_{k}(B^{+})=\max_{k\leq i\leq n} \{ \frac{1}{\vert b_{ii}\vert } \sum_{j=k, \neq i}^{n} \vert b_{ij}\vert \}$ and

$$\prod_{j=1}^{i-1} \Biggl(1+\frac{1}{\bar{\beta}_{j}} \sum_{k=j+1}^{n} \vert b_{jk} \vert \Biggr)=1, \quad\mbox{if } i=1. $$

Recently, when *M* is a weakly chained diagonally dominant (*wcdd*) *B*-matrix, Li *et al.* [13] gave a bound for $\max_{d\in[0,1]^{n}} \Vert (I-D+DM)^{-1}\Vert _{\infty }$: Let $M=[m_{ij}]\in\mathbb{R}^{n\times n}$ be a *wcdd* *B*-matrix with the form $M=B^{+}+C$, where $B^{+}=[b_{ij}]$ is defined as (1). Then

4 $$ \max_{d\in[0,1]^{n}} \bigl\Vert (I-D+DM)^{-1}\bigr\Vert _{\infty }\leq \sum_{i=1}^{n} \Biggl( \frac{n-1}{\min\{\tilde{\beta}_{i},1\}} \prod_{j=1}^{i-1} \frac{b_{jj}}{\tilde{\beta}_{j}} \Biggr), $$

where $\tilde{\beta}_{i}=b_{ii}-\sum_{j= i+1}^{n} \vert b_{ij}\vert >0$ and $\prod_{j=1}^{i-1}\frac{b_{jj}}{\tilde{\beta}_{j}}=1$ if $i=1$.

This bound in (4) holds when *M* is a *B*-matrix since a *B*-matrix is a weakly chained diagonally dominant *B*-matrix [13].

Now, some notation is given, which will be used in the sequel. Let $A=[a_{ij}]\in\mathbb{R}^{n\times n}$. For $i, j, k \in \mathbb{N}$, denote

$$\begin{aligned} &u_{i}(A)=\frac{1}{\vert a_{ii}\vert } \sum _{j= i+1}^{n} \vert a_{ij}\vert , \qquad u_{n}(A)=0, \\ &b_{k}(A)= \max_{k+1\leq i\leq n} \biggl\{ \frac{\sum_{j= k, \neq i}^{n} \vert a_{ij}\vert }{\vert a_{ii}\vert } \biggr\} , \qquad b_{n}(A)=1, \\ &p_{k}(A)= \max_{k+1\leq i\leq n} \biggl\{ \frac{ \vert a_{ik}\vert +\sum_{j= k+1, \neq i}^{n} \vert a_{ij}\vert b_{k}(A) }{\vert a_{ii}\vert } \biggr\} , \qquad p_{n}(A)=1. \end{aligned} $$

The rest of this paper is organized as follows: In Section 2, we present some new bounds for $\max_{d\in[0,1]^{n}} \Vert (I-D+DM)^{-1}\Vert_{\infty}$ when *M* is a *wcdd* *B*-matrix. Numerical examples are given to verify the corresponding results in Section 3.

## Main results

In this section, some new upper bounds for $\max_{d\in [0,1]^{n}} \Vert (I-D+DM)^{-1}\Vert _{\infty}$ are provided when *M* is a *wcdd* *B*-matrix. Firstly, several lemmas, which will be used later, are given.

### Lemma 1

[13]

*Let* $M=[m_{ij}]\in\mathbb{R}^{n\times n}$ *be a wcdd* *B*-*matrix with the form* $M=B^{+}+C$, *where* $B^{+}$ *is defined as* (1). *Then*

$$\bigl\Vert \bigl(I + \bigl(B^{+}_{D}\bigr)^{-1}C_{D} \bigr)^{-1}\bigr\Vert _{\infty} \leq n-1, $$

*where* $B^{+}_{D}=I-D+DB^{+}$ *and* $C_{D}=DC$.

### Lemma 2

[15]

*Let* $A=[a_{ij}]\in\mathbb{R}^{n\times n}$ *be a wcdd* *M*-*matrix with* $u_{k}(A)p_{k}(A)<1$ ($\forall k\in\mathbb{N}$). *Then*

$$\begin{aligned} \bigl\Vert A^{-1}\bigr\Vert _{\infty} &\leq \max\Biggl\{ \sum_{i=1}^{n} \Biggl( \frac{1}{ a_{ii} (1-u_{i}(A)p_{i}(A) ) } \prod_{j=1}^{i-1} \frac{u_{j}(A)}{1-u_{j}(A)p_{j}(A)} \Biggr), \\ &\quad \sum_{i=1}^{n} \Biggl( \frac{p_{i}(A)}{ a_{ii} (1-u_{i}(A)p_{i}(A) ) } \prod_{j=1}^{i-1} \frac{1}{1-u_{j}(A)p_{j}(A)} \Biggr) \Biggr\} , \end{aligned} $$

*where*

$$\prod_{j=1}^{i-1} \frac{u_{j}(A)}{1-u_{j}(A)p_{j}(A)} =1, \qquad \prod_{j=1}^{i-1} \frac{1}{1-u_{j}(A)p_{j}(A)}=1, \quad\textit{if } i=1. $$

### Lemma 3

[14]

*Let* $\gamma>0$ *and* $\eta\geq0$. *Then*, *for any* $x\in[0,1]$,

$$\frac{1}{1-x+\gamma x}\leq \frac{1}{ \min\{\gamma, 1 \}}, \qquad \frac{\eta x}{1-x+\gamma x}\leq \frac{\eta}{\gamma}. $$

### Theorem 1

*Let* $M=[m_{ij}]\in\mathbb{R}^{n\times n}$ *be a wcdd* *B*-*matrix with the form* $M=B^{+}+C$, *where* $B^{+}=[b_{ij}]$ *is defined as* (1). *If*, *for each* $i\in\mathbb{N}$,

$$\hat{\beta}_{i}=b_{ii}-\sum_{j= i+1}^{n} \vert b_{ij}\vert p_{i}\bigl(B^{+}\bigr)>0, $$

*then*

5 $$ \begin{aligned}[b] &\max_{d\in[0,1]^{n}} \bigl\Vert (I-D+DM)^{-1}\bigr\Vert _{\infty } \\ &\quad\leq\max \Biggl\{ \sum_{i=1}^{n} \frac{n-1}{ \min \{\hat{\beta}_{i}, 1\} } \prod_{j=1}^{i-1} \Biggl( \frac{1}{\hat{\beta}_{j} }\sum_{k= j+1}^{n} \vert b_{jk}\vert \Biggr), \sum_{i=1}^{n} \frac{(n-1)p_{i}(B^{+})}{ \min \{\hat{\beta}_{i}, 1\} } \prod_{j=1}^{i-1} \frac{ b_{jj} }{\hat{\beta}_{j} } \Biggr\} , \end{aligned} $$

*where*

$$\prod_{j=1}^{i-1} \Biggl(\frac{1}{\hat{\beta}_{j} } \sum_{k= j+1}^{n} \vert b_{jk} \vert \Biggr) =1, \qquad \prod_{j=1}^{i-1} \frac{ b_{jj} }{\hat{\beta}_{j} }=1, \quad \textit{if } i=1. $$

### Proof

Let $M_{D}=I-D+DM$. Then

$$M_{D}=I-D+DM=I-D+D\bigl(B^{+}+C\bigr)=B_{D}^{+}+C_{D}, $$

where $B_{D}^{+}=I-D+DB^{+}$. Similar to the proof of Theorem 2 in [13], we see that $B_{D}^{+}$ is a *wcdd* *M*-matrix with positive diagonal elements and $C_{D}=DC$, and, by Lemma 1,

6 $$ \bigl\Vert M_{D}^{-1}\bigr\Vert _{\infty}\leq \bigl\Vert \bigl(I+\bigl(B_{D}^{+}\bigr)^{-1}C_{D} \bigr)^{-1}\bigr\Vert _{\infty} \bigl\Vert \bigl(B_{D}^{+} \bigr)^{-1}\bigr\Vert _{\infty} \leq (n-1)\bigl\Vert \bigl(B_{D}^{+}\bigr)^{-1}\bigr\Vert _{\infty}. $$

By Lemma 2, we have

$$\begin{aligned} \bigl\Vert \bigl(B_{D}^{+} \bigr)^{-1}\bigr\Vert _{\infty} &\leq \max\Biggl\{ \sum_{i=1}^{n} \frac{1}{ (1-d_{i}+b_{ii}d_{i}) (1-u_{i}(B_{D}^{+})p_{i}(B_{D}^{+}) ) } \prod_{j=1}^{i-1} \frac{u_{j}((B_{D}^{+}))}{1-u_{j}((B_{D}^{+}))p_{j}(B_{D}^{+})}, \\ &\quad \sum_{i=1}^{n} \frac{p_{i}(B_{D}^{+})}{ (1-d_{i}+b_{ii}d_{i}) (1-u_{i}((B_{D}^{+}))p_{i}(B_{D}^{+}) ) } \prod_{j=1}^{i-1} \frac{1}{1-u_{j}(B_{D}^{+})p_{j}(B_{D}^{+})} \Biggr\} . \end{aligned} $$

By Lemma 3, we can easily get the following results: for each $i , j, k\in\mathbb{N}$,

$$\begin{aligned}& b_{k}\bigl(B_{D}^{+} \bigr)= \max_{k+1\leq i\leq n} \biggl\{ \frac{\sum_{j= k, \neq i}^{n} \vert b_{ij}\vert d_{i} }{1-d_{i}+b_{ii}d_{i}} \biggr\} \leq \max _{k+1\leq i\leq n} \biggl\{ \frac{\sum_{j= k, \neq i}^{n} \vert b_{ij}\vert }{b_{ii}} \biggr\} =b_{k} \bigl(B^{+}\bigr), \\& p_{k}\bigl(B_{D}^{+}\bigr)= \max _{k+1\leq i\leq n} \biggl\{ \frac{ \vert b_{ik}\vert d_{i}+\sum_{j= k+1, \neq i}^{n} \vert b_{ij}\vert d_{i}b_{k}(B_{D}^{+}) }{1-d_{i}+b_{ii}d_{i}} \biggr\} \\& \hphantom{p_{k}(B_{D}^{+})}\leq\max_{k+1\leq i\leq n} \biggl\{ \frac{ \vert b_{ik}\vert +\sum_{j= k+1, \neq i}^{n} \vert b_{ij}\vert b_{k}(B_{D}^{+}) }{b_{ii}} \biggr\} \\& \hphantom{p_{k}(B_{D}^{+})} \leq\max_{k+1\leq i\leq n} \biggl\{ \frac{ \vert b_{ik}\vert +\sum_{j= k+1, \neq i}^{n} \vert b_{ij}\vert b_{k}(B^{+}) }{b_{ii}} \biggr\} \\& \hphantom{p_{k}(B_{D}^{+})}=p_{k}\bigl(B^{+}\bigr), \end{aligned}$$

and

7 $$ \begin{aligned}[b] \frac{1}{ (1-d_{i}+b_{ii}d_{i})(1-u_{i}(B_{D}^{+})p_{i}(B_{D}^{+}))} &= \frac{1}{ 1-d_{i}+b_{ii}d_{i}-\sum_{j= i+1}^{n} \vert b_{ij}\vert d_{i} p_{i}(B_{D}^{+})} \\ &\leq\frac{1}{ \min \{ b_{ii}-\sum_{j= i+1}^{n} \vert b_{ij}\vert p_{i}(B^{+}), 1 \} } \\ &=\frac{1}{ \min \{ \hat{\beta}_{i}, 1 \} }. \end{aligned} $$

Furthermore, by Lemma 3, we have

8 $$ \begin{aligned}[b] \frac{u_{i}(B_{D}^{+})}{ 1-u_{i}(B_{D}^{+})p_{i}(B_{D}^{+})} &= \frac{\sum_{j= i+1}^{n} \vert b_{ij}\vert d_{i} }{ 1-d_{i}+b_{ii}d_{i} -\sum_{j=i+1}^{n} \vert b_{ij}\vert d_{i} p_{i}(B_{D}^{+})} \\ &\leq\frac{\sum_{j= i+1}^{n} \vert b_{ij}\vert }{ b_{ii}-\sum_{j= i+1}^{n} \vert b_{ij}\vert p_{i}(B^{+}) } \\ &=\frac{1}{\hat{\beta}_{i} }\sum_{j= i+1}^{n} \vert b_{ij}\vert \end{aligned} $$

and

9 $$ \begin{aligned}[b] \frac{1}{ 1-u_{i}(B_{D}^{+})p_{i}(B_{D}^{+})} &= \frac{1-d_{i}+b_{ii}d_{i} }{ 1-d_{i}+b_{ii}d_{i} -\sum_{j=i+1}^{n} \vert b_{ij}\vert d_{i} p_{i}(B_{D}^{+})} \\ &\leq\frac{1-d_{i}+b_{ii}d_{i} }{ b_{ii}-\sum_{j= i+1}^{n} \vert b_{ij}\vert p_{i}(B^{+}) } \\ &=\frac{b_{ii}}{\hat{\beta}_{i} }. \end{aligned} $$

By (7), (8), and (9), we obtain

10 $$ \bigl\Vert \bigl(B_{D}^{+} \bigr)^{-1}\bigr\Vert _{\infty} \leq\max \Biggl\{ \sum _{i=1}^{n} \frac{1}{ \min \{\hat{\beta}_{i}, 1\} } \prod _{j=1}^{i-1} \Biggl(\frac{1}{\hat{\beta}_{j} }\sum _{k= j+1}^{n} \vert b_{jk}\vert \Biggr), \sum_{i=1}^{n} \frac {p_{i}(B^{+})}{ \min \{\hat{\beta}_{i}, 1\} } \prod _{j=1}^{i-1} \frac{ b_{jj} }{\hat{\beta}_{j} } \Biggr\} . $$

Therefore, the result in (5) holds from (6) and (10). □

Since a *B*-matrix is also a *wcdd* *B*-matrix, then by Theorem 1, we find the following result.

### Corollary 1

*Let* $M=[m_{ij}]\in\mathbb{R}^{n\times n}$ *be a* *B*-*matrix with the form* $M=B^{+}+C$, *where* $B^{+}=[b_{ij}]$ *is defined as* (1). *Then*

11 $$ \begin{aligned}[b] & \max_{d\in[0,1]^{n}} \bigl\Vert (I-D+DM)^{-1}\bigr\Vert _{\infty} \\ &\quad\leq \max \Biggl\{ \sum_{i=1}^{n} \frac{n-1}{ \min \{\hat{\beta}_{i}, 1\} } \prod_{j=1}^{i-1} \Biggl( \frac{1}{\hat{\beta}_{j} }\sum_{k= j+1}^{n} \vert b_{jk}\vert \Biggr), \sum_{i=1}^{n} \frac{(n-1)p_{i}(B^{+})}{ \min \{\hat{\beta}_{i}, 1\} } \prod_{j=1}^{i-1} \frac{ b_{jj} }{\hat{\beta}_{j} } \Biggr\} , \end{aligned} $$

*where* $\hat{\beta}_{i}$ *is defined as in Theorem* 1.

We next give a comparison of the bounds in (4) and (5) as follows.

### Theorem 2

*Let* $M=[m_{ij}]\in\mathbb{R}^{n\times n}$ *be a wcdd* *B*-*matrix with the form* $M=B^{+}+C$, *where* $B^{+}=[b_{ij}]$ *is defined as* (1). *Let* $\bar{\beta}_{i}$, $\tilde{\beta}_{i}$, *and* $\hat{\beta}_{i}$ *be defined as in* (3), (4), *and* (5), *respectively*. *Then*

12 $$ \begin{aligned}[b] &\max \Biggl\{ \sum _{i=1}^{n} \frac{n-1}{ \min \{\hat{\beta}_{i}, 1\} } \prod _{j=1}^{i-1} \Biggl(\frac{1}{\hat{\beta}_{j} }\sum _{k= j+1}^{n} \vert b_{jk}\vert \Biggr), \sum_{i=1}^{n} \frac{(n-1)p_{i}(B^{+})}{ \min \{\hat{\beta}_{i}, 1\} } \prod _{j=1}^{i-1} \frac{ b_{jj} }{\hat{\beta}_{j} } \Biggr\} \\ &\quad \leq \sum_{i=1}^{n} \Biggl( \frac{n-1}{\min\{\tilde{\beta}_{i},1\}} \prod_{j=1}^{i-1} \frac{b_{jj}}{\tilde{\beta}_{j}} \Biggr). \end{aligned} $$

### Proof

Since $B^{+}$ is a *wcdd* matrix with positive diagonal elements, for any $i\in\mathbb{N}$,

13 $$ 0\leq p_{i}\bigl(B^{+}\bigr)\leq1, \qquad \tilde{\beta}_{i}\leq \hat{\beta}_{i}. $$

By (13), for each $i\in\mathbb{N}$,

14 $$ \frac{1}{ \hat{\beta}_{i} } \leq\frac{1}{ \tilde{\beta}_{i} }, \qquad \frac{1}{ \min\{ \hat{\beta}_{i}, 1\} } \leq\frac{1}{ \min \{ \tilde{\beta}_{i}, 1\} }. $$

The result in (12) follows by (13) and (14). □

### Remark 1

(i) Theorem 2 shows that the bound in (5) is better than that in (4).
(ii) When *n* is very large, one needs more computations to obtain these upper bounds by (5) than by (4).

## Numerical examples

In this section, we present numerical examples to illustrate the advantages of our derived results.

### Example 1

Consider the family of *B*-matrices in [14]:

$$M_{k}=\left [ \begin{matrix} 1.5 &0.5 &0.4 &0.5 \\ -0.1 &1.7 &0.7 &0.6 \\ 0.8 &-0.1 \frac{k}{k+1} &1.8 &0.7\\ 0 &0.7 &0.8 &1.8 \end{matrix} \right ], $$

where $k\geq1$. Then $M_{k}=B_{k}^{+}+C_{k}$, where

$$B_{k}^{+}=\left [ \begin{matrix} 1 &0 &-0.1 &0 \\ -0.8 &1 &0 &-0.1 \\ 0 &-0.1 \frac{k}{k+1}-0.8 &1 &-0.1\\ -0.8 &-0.1 &0 &1 \end{matrix} \right ]. $$

By (2), we have

$$\max_{d\in[0,1]^{4}} \bigl\Vert (I-D+DM_{k})^{-1} \bigr\Vert _{\infty }\leq \frac{4-1}{\min\{\beta,1\}}=30(k+1). $$

It is obvious that

$$30(k+1)\rightarrow+\infty, \quad \mbox{if } k\rightarrow+\infty. $$

By (3), we get

$$\max_{d\in[0,1]^{4}} \bigl\Vert (I-D+DM_{k})^{-1} \bigr\Vert _{\infty }\leq 15.2675. $$

By Theorem 7 of [11], we have

$$\max_{d\in[0,1]^{4}} \bigl\Vert (I-D+DM_{k})^{-1} \bigr\Vert _{\infty }\leq 13.6777. $$

By Corollary 1 of [13], we have

$$\max_{d\in[0,1]^{4}} \bigl\Vert (I-D+DM_{k})^{-1} \bigr\Vert _{\infty }\leq \sum_{i=1}^{4} \Biggl(\frac{3}{\min\{\tilde{\beta}_{i},1\}} \prod_{j=1}^{i-1} \frac{b_{jj}}{\tilde{\beta}_{j}} \Biggr)\approx15.2675. $$

By (11), we obtain

$$\max_{d\in[0,1]^{4}} \bigl\Vert (I-D+DM_{k})^{-1} \bigr\Vert _{\infty }\leq 9.9683. $$

In these two cases, the bounds in (2) are equal to 60 ($k=1$) and 90 ($k=2$), respectively.

### Example 2

Consider the *wcdd* *B*-matrix in [13]:

$$M=\left [ \begin{matrix} 1.5 &0.2 &0.4 &0.5 \\ -0.1 &1.5 &0.5 &0.1 \\ 0.5 &-0.1 &1.5 &0.1\\ 0.4 &0.4 &0.8 &1.8 \end{matrix} \right ]. $$

Then $M=B^{+}+C$, where

$$B^{+}=\left [ \begin{matrix} 1 &-0.3 &-0.1 &0 \\ -0.6 &1 &0 &-0.4 \\ 0 &-0.6 &1 &-0.4\\ -0.4 &-0.4 &0 &1 \end{matrix} \right ]. $$

By (4), we get

$$\max_{d\in[0,1]^{4}} \bigl\Vert (I-D+DM)^{-1}\bigr\Vert _{\infty }\leq 41.1111. $$

By (5), we have

$$\max_{d\in[0,1]^{4}} \bigl\Vert (I-D+DM)^{-1}\bigr\Vert _{\infty }\leq 21.6667. $$

## Conclusions

In this paper, we present some new upper bounds for $\max_{d\in[0,1]^{n}} \Vert (I-D+DM)^{-1}\Vert _{\infty}$ when *M* is a weakly chained diagonally dominant *B*-matrix, which improve some existing results. A numerical example shows that the given bounds are efficient.
